# Descriptive analysis of genital colonization across gestational trimesters in pregnant women with different obstetric histories

**DOI:** 10.61622/rbgo/2026rbgo29

**Published:** 2026-07-17

**Authors:** Carolina Pereira Andrade, Camila Marconi, Márcia Guimarães da Silva, Newton Sérgio de Carvalho

**Affiliations:** 1 Universidade Federal do Paraná Curitiba PR Brazil Universidade Federal do Paraná Curitiba, PR, Brazil; 2 Universidade Federal do Paraná Curitiba PR Brazil Universidade Federal do Paraná, Curitiba, PR, Brazil; 3 Faculdade de Medicina de Botucatu SP Brazil Faculdade de Medicina de Botucatu, SP, Brazil; 4 Universidade Federal do Paraná Curitiba PR Brazil Universidade Federal do Paraná, Curitiba, PR, Brazil

**Keywords:** Vaginal microbiome, Preterm delivery, Ureaplasma, Mycoplasma hominis, Pregnant women

## Abstract

**Objective:**

The aim of this study was to assess the *Ureaplasma parvum* variation of genital colonization during pregnancy with an exploratory approach among women with term and preterm birth histories.

**Methods:**

Prospective case-control study of 24 pregnant women with history of full-term pregnancy and 26 pregnant women with history of prematurity whose endpoint was to assess the colonization pattern of these agents with a vaginal/cervical sample collected at the beginning of each trimester of gestation. Collected data: sociodemographic characteristics, previous diseases, gynecological and obstetric history, and microorganisms in vaginal secretion samples by real-time polymerase chain reaction (PCR).

**Results:**

The overall number of cases positive for at least one microorganism was 18 (36.0%). A significantly higher positivity rate was observed in the second and third trimesters in both study groups compared with the first trimester (p<0.001). The microorganisms identified in the vaginal environment were *Ureaplasma parvum* (66.6%), *Mycoplasma hominis* (16.7%), and both (16.7%), and the frequency of both these microorganisms was comparable in both groups at each pregnancy trimester (p for all = nonsignificant), but with a difference between the trimesters. Prematurity in the current pregnancy occurred in a single case (6.7%) in a pregnant woman without history of prematurity in the previous pregnancy (p=1.00).

**Conclusion:**

The most frequently identified microorganism was *Ureaplasma parvum*, and the overall incidence of positive cases including all microorganisms was 36.0%. Although *Ureaplasma parvum* was the most frequently identified microorganism, the sample was not powered to assess its association with prematurity.

## Introduction

Premature birth is one of the leading causes of neonatal morbidity and mortality worldwide. *Ureaplasma* spp. and *Mycoplasma* spp. have been frequently isolated from amniotic fluid and placenta in preterm births and are associated with reports of infertility, stillbirth, chorioamnionitis, and neonatal and perinatal morbidity.^(1)^

Although almost 50 years have passed since these microorganisms have been associated with perinatal and neonatal complications, the pathological effects, and distinctions between the various species and biovars of both *Mycoplasma* and *Ureaplasma* remain unclear. The emergence of molecular-based investigations has revitalized the research on these microorganisms, allowing a better understanding of their pathogenesis, especially regarding their transition from commensalism to infection.^(2)^

*Mycoplasma* spp. and *Ureaplasma* spp. were initially studied using enzyme-linked immunosorbent assay (ELISA) techniques, but it was only after advancements in DNA amplification techniques by real-time PCR that these microorganisms became better detected and identified.^(2)^

Studies reveal that the rates of genital colonization by these microorganisms are comparable worldwide, ranging from 18–51% and 51–80%, respectively,^(2,3)^ and although many studies have found associations between colonization/infection and unfavorable pregnancy and neonatal, perinatal, and postpartum outcomes, systematic reviews, and meta-analyses published in the last 12 years^(4,5)^ and recent studies^(6,7)^ have shown conflicting results in this regard. The challenges in establishing such cause-effect relationship reside in the following factors: 1) need for accuracy in differentiating the presence of the bacteria as commensal or a pathogen, 2) occurrence of a polymicrobial nature in the vaginal microbiota, 3) difficulty in differentiating *Ureaplasma* spp. from other microorganisms, and 4) presence of other factors involved in the pathophysiology of this complex disorder.^(8)^

This study was conducted to assess the variation of genital colonization during pregnancy with an exploratory approach among women with term and preterm birth histories.

## Methods

**Figure 1:**
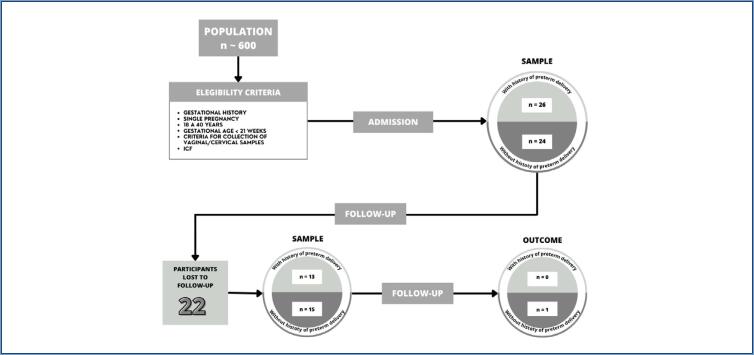
Flowchart illustrating study participants

Source: Flow diagram constructed according to STROBE recommendations.^(9)^

Case-control study conducted at a low-risk prenatal service in a basic public health care unit in southern Brazil from July 2018 to February 2020. 50 pregnant women were enrolled according to their subsequent order of admission to the service for routine prenatal consultations. Inclusion criteria: a) with or without history of preterm delivery/abortion, b) age between 18 and 40 years, c) single pregnancy, d) gestational age of up to 20 weeks at the first prenatal visit, e) fulfillment of criteria for collection of vaginal/cervical material (absence of antimicrobial therapy in the last 30 days, 72 hours of sexual abstinence, 72 hours of any vaginal procedure, such as vaginal examination and vaginal ultrasound), and f) signed free and informed consent form. Exclusion criteria: comorbidities, and a positive test for *Chlamydia trachomatis, Trichomonas vaginalis*, or *Neisseria gonorrhoeae*. Patients diagnosed with any infection were managed according to the national prenatal care protocol. The participants without history of preterm delivery were allocated to the full-term (FT) group (n = 24), while those with history of preterm delivery comprised the preterm (PT) group (n = 26). In all, 28 pregnant women were followed up during the three pregnancy trimesters, 15 in the FT group and 13 in the PT group. Two patients tested positive for *Chlamydia trachomatis* by nucleic acid amplification tests (NAATs) performed on vaginal/cervical swabs collected at study enrollment and were excluded as they required treatment, while 20 patients did not return for further evaluation (Figure 1).

All participants answered a questionnaire collecting information about sociodemographic data, lifestyle habits, gynecological and obstetric history, type of delivery, and newborn weight. Also underwent routine obstetric evaluation and gynecological examination for collection of vaginal/cervical samples over three prenatal consultations, one in each pregnancy trimester. Vaginal pH was measured, and samples were collected from the middle third of the vaginal wall using sterile swabs and from the cervix. The collected material was used for preparation of vaginal smears for classification of the vaginal microbiota and detection of infections by *Trichomonas vaginalis, Chlamydia trachomatis*, and *Neisseria gonorrhoeae*, and the presence of the microorganisms, *Mycoplasma genitalium*, *Mycoplasma hominis, Ureaplasma urealyticum*, and *Ureaplasma parvum* using nucleic acid amplification tests (NAATs), as recommended for high-sensitivity screening.^(10)^

The vaginal microbiota was classified into one of the following categories: a) normal (scores 0 to 3), b) intermediate (scores 4 to 6, represented by the presence of morphotypes compatible with *Lactobacillus* concomitant with an expressive accessory microbiota), and c) bacterial vaginosis (scores 7 to 10, represented by depletion of *Lactobacillus* and replacement by granular microbiota). A search for morphotypes compatible with pseudohyphae or hyphae of *Candida* spp. was also performed.^(11)^

The results are reported following the STrengthening the Reporting of Observational Studies in Epidemiology (STROBE) guidelines.^(9)^

Measures of central tendency and dispersion are expressed as mean and standard deviation (SD) for continuous variables with symmetric distribution and as median and interquartile range (IQR) for those with asymmetric distribution. Categorical variables are expressed as absolute and relative frequency.

The Student's *t* test was applied to estimate differences between continuous variables with symmetric distribution, while the Mann-Whitney test was used for those with asymmetric distribution. Fisher's exact and Pearson's chi-square tests were applied to estimate differences between categorical variables. A significance level of 5% was considered for all tests.

To determine the required sample size to detect a 45% difference between trimesters, we use the formula for comparing proportions:


$$n=\frac{Z\alpha \frac{1}{2}\cdot \sqrt{2p(1-p)}+Z\beta \cdot {\sqrt{p1(1-p1)+p2(1-p2)}}^{2}}{(p1-p2{)}^{2}}$$

Confidence level: 95% → Zα/2=1.96; statistical power: 80% → Zβ=0.84

Expected proportions: p1=0.50 (initial assumed proportion); p2=0.05

The required sample size was 15 participants per group without adjusting for losses. Considering a 20% loss rate, the adjusted sample size increases to 19 participants per group. For the follow-up, the estimated sample was 15 pregnant women in each group.

The study was approved by the Human Research Ethics Committee of the Health Sciences Sector of the Federal University of Paraná with the number #CAAE 57341416.3.3002.0096.

## Results

The 50 participants included in the study had a mean age of 29.4±5.9 years and a mean gestational age of 13.6±4.5 weeks. Participants in the PT group had a younger maternal age than those in the FT group (p<0.001). The groups were similar in terms of tobacco, alcohol, and drug use, number of partners, and number of sexual intercourses per week (Table 1).

**Table 1:** Sociodemographic characteristics of the sample (n = 50)

Characteristics	FT (n=24) n (%)	PT (n=26) n(%)	p-value
Age (years) (mean+SD)	31.7±5.3	27.4±5.7	*<0.001
Ethnicity			
Caucasian	13(56.5)	19(73.1)	0.24
Black and mixed race	10(43.5)	7(26.9)	
Exercising a professional activity	14(58.3)	12(46.1)	0.41
Other family income contribution	22(91.7)	21(81.0)	0.44
Monthly income (in Brazilian Real) [median (IQR)]	2500(1700-4000)	2500(2000-3500)	0.12
People living on full income			
1	3(12.5)	2(7.7)	0.64
2	4(16.7)	2(7.7)	
≥ 3	17(70.1)	22(84.6)	
Smoking	2(8.3)	4(19.2)	0.58
Alcoholism	2(8.3)	2(7.7)	1.00
Drug addiction	0(0.0)	2(8.0)	0.48
Lives with partner	22(91.7)	24(92.3)	1.00
New partner	3(12.5)	4(15.4)	1.00
Number of partners			
1	23(95.8)	1(4.2)	1.00
2	18(100.0)	0(0.0)	
Number of sexual relations per week			
1-2	16(66.7)	13(50.0)	0.42
3-4	6(25.0)	13(50.0)	
5-7	2(8.3)	0(0.0)	

* Significant difference (p< 0.05): comparison of FT and PT groups; Student's *t* test, Fisher's exact test, Mann-Whitney test or Pearson's chi-square test for differences between FT and PT groups. FT - group without history of preterm delivery; PT - group with history of preterm delivery; SD - standard deviation; IQR - interquartile range.

History of sexually transmitted infection, genital complaints, vaginal pH, and classification of the genital microbiota were also comparable between the groups (p for all=nonsignificant). The PT group had a higher gestational age (p=0.02), a greater number of pregnancies (p=0.03), and history of more abortions (p<0.001), which was expected considering that previous abortion was a criterion for inclusion of the participant in the PT group. No difference was observed between groups regarding the type of delivery (p=1.00), newborn weight (p=0.60), and frequency of colonization by *Candida* spp, *Chlamydia trachomatis, Mycoplasma hominis*, or *Ureaplasma parvum* (p for all = nonsignificant). There was also no difference regarding Nugent scores or frequency of positivity for *Mycoplasma hominis* or *Ureaplasma parvum* in the three trimesters (p for all=nonsignificant) (Table 2).

**Table 2:** History of sexually transmitted infection, cervical cancer screening, genital complaints, and classification of vaginal microbiota

Variables		FT (n=24) n(%)	PT (n=26) n(%)	p-value
History of sexually transmitted infection		2(8.3)	1(3.8)	0.60
History of alteration in vaginal content		12(50.0)	14(56.0)	0.91
Pap test**		20(100.0)	18(90.0)	0.48
Complaint of discharge		5(20.8)	7(26.9)	0.70
Complaint of foul genital odor		3(12.5)	5(19.2)	0.43
Complaint of genital itching		0(0.0)	1(3.8)	1.00
Vaginal pH (mean+SD)		4.24±0.30	4.15±0.25	0.27
*Candida* spp. microscopy		2(8.3)	2(7.7)	1.00
Nugent classification				
	0-3	21(87.5)	20(76.9)	
	4-6	0(0.0)	0(0.0)	0.48
	7-10	2(8.3)	5(19.2)	
Gestational age (mean+SD)		12.2±3.5	14.9±4.96	*0.02
Ultrasonography		14(63.6)	19(90.5)	0.06
LMP		8(36.4)	2(9.5)	
Number of pregnancies				
	2	18(75.0)	8(30.8)	*0.01
	3	6(25.0)	10(38.5)	
	> 4	0(0.0)	8(30.8)	
Number of deliveries				
	0	0(0.0)	1(3.8)	0.16
	1	18(75.0)	14(53.8)	
	2	6(25.0)	6(23.1)	
	> 3	0(0.0)	5(19.2)	
Number of abortions				
	0	24(100.0)	16(61.5)	
	1	0(0.0)	9(34.6)	*<0.001
	2	0(0.0)	1(3.8)	
Type of delivery				
	Normal	12(57.1)	14(42.9)	1.00
	C-section	9(42.9)	11(44.0)	
Newborn weight (mean+SD)		3188.5±477.3	3088.0±420.0	0.60
PCR for detection of cervical infection		4(16.7)	5(19.2)	1.00
*Chlamydia trachomatis*		2(8.3)	2(7.7)	1.00
*Mycoplasma hominis*		2(8.3)	0(0.0)	0.22
*Ureaplasma parvum*		1(4.2)	4(15.4)	0.35
*Mycoplasma genitalium*		0(0.0)	0(0.0)	1.00
*Ureaplasma urealyticum*		0(0.0)	0(0.0)	1.00
*Candida* spp. Culture**		3(12.5)	5(19.2)	0.44

* Significant difference (p< 0.05): comparison of FT and PT groups; Fisher's exact test, Student's *t* test, or Pearson's chi-square test for differences between FT and PT groups.

** FT: n = 20; PT: n = 14; LMP - last menstrual period; FT - group without history of preterm delivery; PT - group with history of preterm delivery; SD - standard deviation.

**Figure 2:**
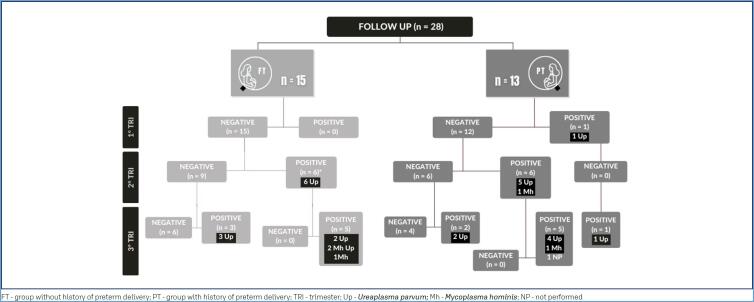
Flowchart of the cases positive for *Ureaplasma parvum* and *Mycoplasma hominis* in pregnancy

Between the participants who completed all three follow-up visits (n=28) and those who were lost to follow up (n=22), no significant differences were observed in the analyzed variables (p for all = nonsignificant). The overall incidence of positivity for the microorganisms – considering all participants with a positive result in the three trimesters and two participants who were positive in the 2nd trimester but negative in the 3rd trimester – was 36.0% (n=18). Among the participants in the PT group, only one was positive for *Ureaplasma parvum* in the first trimester; this participant had, for the same microorganism, a negative result in the second trimester and, again, a positive result in the third trimester. The other participants in the PT group had a negative test in the first trimester; six of them tested positive in the second trimester (five for *Ureaplasma parvum* and one for *Mycoplasma hominis*). Of the five participants who tested positive for *Ureaplasma parvum* in the second trimester, four remained positive and one was lost to follow up in the third trimester. The only patient who tested positive for *Mycoplasma hominis* in the second semester remained positive for this microorganism in the third trimester (Figure 2).

There was a significant increase in tests positive for microorganisms in the vaginal environment, from one case in the first trimester (3.6%) to 12 cases in the second trimester (3.6% versus 42.8%, p<0.001) and 16 cases in the third trimester (3.6% versus 61.5%; p<0.001) (Table 3).

**Table 3:** Number and total of positive cases for microorganisms according to the trimester of pregnancy

Microorganisms	FT (n = 15) [n (%)]			p-value	PT (n = 13) [n (%)]			p-value
	Trimester				Trimester			
	First	Second	Third		First	Second	Third	
*Mycoplasma hominis*	0(0.0)	0(0.0)	3(20.0)	0.22	0(0.0)	1(7.7)	1(7.7)	0.96
*Ureaplasma parvum*	0(0.0)	6(40.0)	7(46.7)	*<0.01	1(7.7)	5(38.5)	7(53.8)	**0.03
Total	0(0.0)	6(40.0)	10(66.7)	*<0.01	1(7.7)	6(46.1)	8(61.5)	**0.01

* Significant difference (p< 0.05) comparison of FT and PT groups for first versus second trimester/first versus third trimester

** Significant difference (p < 0.05): comparison of FT and PT groups for first versus third trimester. Fisher's exact test for differences between FT and PT groups. FT - group without history of preterm delivery; PT - group with history of preterm delivery

The frequency of tests positive in all three trimesters was similar in both groups, and no significant differences were observed based on the presence or absence of history of preterm delivery (p for all = nonsignificant) (Table 3). Only one case of prematurity was observed among all 28 participants who were followed up across all three trimesters (3.6%), and it was recorded in a participant in the FT group (0.0% in the PT group versus 3.6% in the FT group; p=1.00), which had a positive test for *Ureaplasma parvum* in the second trimester and for *Ureaplasma parvum* and *Mycoplasma hominis* in the third trimester. Another case with positivity for *Ureaplasma parvum* and *Mycoplasma hominis* was observed, but in the FT group.

## Discussion

Although differences between groups were explored, the sample size limits the ability to draw conclusions regarding associations with preterm delivery. The highest incidence was observed for *Ureaplasma parvum*, and there was a significant increase in positivity for *Mycoplasma hominis* and *Ureaplasma parvum* throughout pregnancy, particularly in the last two trimesters. The overall incidence of positivity was 36.0% (n=18).

No differences were observed between the groups regarding Nugent scores or frequency of tests with a positive result, and the most frequently detected microorganism was *Ureaplasma* spp., with an accumulated frequency of 86.7% in the FT group and 100.0% in the PT group. Breugelmans et al.^(12)^ also found no association between abnormal vaginal microbiota and preterm birth. Changes in the vaginal microbiota because of the host's immune response, the virulence of the microorganism, or presence of local factors in the lower genital tract may favor the invasion of the genital tract by *Ureaplasma* spp., but the reason why some pregnant women present premature birth when colonized with these microorganisms (albeit asymptomatic) while others do not remain unknown.^(12)^

Another recent study also found no consistent association between infection by these microorganisms and prematurity. Tétu et al.^(7)^ conducted a cohort study including 821 pregnant women who underwent amniocentesis between 14 and 24 weeks and observed preterm delivery (<35 weeks) in 26 women (3.2%), all of whom had negative PCR results for *Mycoplasma* spp.

Although colonization was detected, the limited sample size prevents assessment of a potential relationship with preterm birth.^(12-16)^ The only case of prematurity recorded in the present study was in a participant with a positive result for *Ureaplasma* spp. in the second trimester and for *Ureaplasma* spp. and *Mycoplasma* spp. in the third trimester. The association of several microorganisms, such as bacterial vaginosis with *Ureaplasma* spp., has been cited as a possibility of triggering the mechanism of uterine contractions, although we did not find the citation of the association of *Ureaplasma* spp. and *Mycoplasma* spp. could have a similar effect.^(17)^

*Ureaplasma parvum* was also the most incident in both study groups, which agrees with the findings by Payne et al.^(16)^ and Albert et al.^(18)^ The combination of change in vaginal microbiota and presence of *Ureaplasma* spp. has been found at a frequency of 17.5% and suggested to be an independent factor for prematurity.^(12)^ Notably, the association between *Ureaplasma* spp. and prematurity was not observed in the present study.

Another important aspect was that these microorganisms increased in frequency in the second and third trimesters and were not detected in the first trimester. This suggests that certain characteristics (immunological, hormonal, or other) in each trimester may prevent these microorganisms from developing in early pregnancy but facilitate their colonization or infection in late pregnancy. Chan et al.^(19)^ have shown that depletion of *Lactobacillus* species and greater bacterial diversity leads to increased levels of mannose binding lectin and certain immunoglobulins (IgM and IgG), complement (C3b and C5), and interleukins (IL8, IL6, and IL-1β), increasing the risk of premature birth. These and other authors have proposed that the dysregulation of the response of the innate and adaptive immune system, through the effect of the complement cascade, may be one of the triggers of the microbiota related to preterm delivery.^(20)^

The reasons for the increasing incidence of *Mycoplasma* spp. over the two final trimesters of pregnancy are unclear. Future studies are needed to elucidate the present study's findings and clarify whether this increasing incidence may influence the gestational outcome, particularly in relation to prematurity. This study reveals new insight into the *Mycoplasma* spp. vaginal pattern during the pregnancy and could bring an important implication factor for future studies exploring relationships between the vaginal microbiome, vaginal host health, and pregnancy outcomes.

The main systematic reviews published to date show inconsistent evidence of association between colonization/infection by *Mycoplasma genitalium* and preterm delivery. These reviews recommend studies using DNA amplification by PCR to elucidate the role of this microorganism in pregnancy and its outcomes since the absence of correct typing could be the actual reason for the existing conflicting results.^(4,5)^

This case-control study carried out to test the hypothesis that colonization of the genital environment by *Ureaplasma* spp. and *Mycoplasma* spp. is related to prematurity in the current pregnancy. The most frequent microorganism identified in our study was *Ureaplasma parvum*. The overall incidence of the microorganisms of interest was 36.0%, with a higher positivity rate in the second and third trimesters. This study was not powered to assess associations between colonization and preterm birth. The reasons for the increasing incidence of *Mycoplasma* spp. over the two final trimesters of pregnancy are unclear. Future studies are needed to elucidate the findings of the present study and clarify whether this increasing incidence may influence the gestational outcome, particularly in relation to prematurity.

## Conclusion

The most frequent microorganism identified in our study was *Ureaplasma parvum*. The overall incidence of the microorganisms was 36.0%, with a higher positivity rate in the second and third trimesters. Although *Ureaplasma parvum* was the most frequently identified microorganism, no definitive conclusions regarding its relationship with prematurity can be drawn due to the limited sample size.

## Data Availability

The research data are described in the article presented.
